# Activity Dependent Degeneration Explains Hub Vulnerability in Alzheimer's Disease

**DOI:** 10.1371/journal.pcbi.1002582

**Published:** 2012-08-16

**Authors:** Willem de Haan, Katherine Mott, Elisabeth C. W. van Straaten, Philip Scheltens, Cornelis J. Stam

**Affiliations:** 1 Department of Clinical Neurophysiology and MEG, VU University Medical Center, Amsterdam, The Netherlands; 2 Alzheimer Center, Department of Neurology, VU University Medical Center, Amsterdam, The Netherlands; Indiana University, United States of America

## Abstract

Brain connectivity studies have revealed that highly connected ‘hub’ regions are particularly vulnerable to Alzheimer pathology: they show marked amyloid-β deposition at an early stage. Recently, excessive local neuronal activity has been shown to increase amyloid deposition. In this study we use a computational model to test the hypothesis that hub regions possess the highest level of activity and that hub vulnerability in Alzheimer's disease is due to this feature. Cortical brain regions were modeled as neural masses, each describing the average activity (spike density and spectral power) of a large number of interconnected excitatory and inhibitory neurons. The large-scale network consisted of 78 neural masses, connected according to a human DTI-based cortical topology. Spike density and spectral power were positively correlated with structural and functional node degrees, confirming the high activity of hub regions, also offering a possible explanation for high resting state Default Mode Network activity. ‘Activity dependent degeneration’ (ADD) was simulated by lowering synaptic strength as a function of the spike density of the main excitatory neurons, and compared to random degeneration. Resulting structural and functional network changes were assessed with graph theoretical analysis. Effects of ADD included oscillatory slowing, loss of spectral power and long-range synchronization, hub vulnerability, and disrupted functional network topology. Observed transient increases in spike density and functional connectivity match reports in Mild Cognitive Impairment (MCI) patients, and may not be compensatory but pathological. In conclusion, the assumption of excessive neuronal activity leading to degeneration provides a possible explanation for hub vulnerability in Alzheimer's disease, supported by the observed relation between connectivity and activity and the reproduction of several neurophysiologic hallmarks. The insight that neuronal activity might play a causal role in Alzheimer's disease can have implications for early detection and interventional strategies.

## Author Summary

An intriguing recent observation is that deposition of the amyloid-β protein, one of the hallmarks of Alzheimer's disease, mainly occurs in brain regions that are highly connected to other regions. To test the hypothesis that these ‘hub’ regions are more vulnerable due to a higher neuronal activity level, we examined the relation between brain connectivity and activity in a computational model of the human brain. Furthermore, we simulated progressive damage to brain regions based on their level of activity, and investigated its effect on the structure and dynamics of the remaining brain network. We show that brain hub regions are indeed the most active ones, and that by damaging networks according to regional activity levels, we can reproduce not only hub vulnerability but a range of phenomena encountered in actual neurophysiological data of Alzheimer patients as well: loss and slowing of brain activity in Alzheimer, loss of synchronization between areas, and similar changes in functional network organization. The results of this study suggest that excessive, connectivity dependent neuronal activity plays a role in the development of Alzheimer, and that the further investigation of factors regulating regional brain activity might help detect, elucidate and counter the disease mechanism.

## Introduction

Like many other complex networks, the human brain contains parts that are better connected to the rest than others: ‘hub’ regions. Evidence is increasing that a collection of brain hub regions forms a ‘structural core’ or ‘connectivity backbone’ that facilitates cognitive processing [1], [2], [3]. Brain hub regions are mainly located in heteromodal association cortices (which integrate information coming from primary cortices), and show a striking overlap with the Default Mode Network [4], [5]. Furthermore, their function has been related to fundamental cognitive features such as consciousness, memory, and IQ [6]–[10]. The central role and large responsibility of hub network regions has an obvious downside: hub damage can have a dramatic impact on network integrity [11], [12]. One of the most intriguing recent insights in this regard has emerged from network-related studies in the field of Alzheimer's disease (AD): cortical hub areas turn out to be exceptionally vulnerable to amyloid deposition, hypometabolism and, eventually, atrophy [13]–[15]. This fascinating link between connectivity and susceptibility to AD pathology deserves further study: what could be causing the hub vulnerability?

The prevailing amyloid-cascade hypothesis of AD states that interstitial amyloid-beta proteins exert a toxic effect on surrounding neurons and synapses, thereby disturbing their function and eventually causing dementia [16]. However, this theory does not provide an explanation for the selective vulnerability of highly connected hub areas. Could an activity-driven mechanism, i.e. hub areas suffering most damage *due to* their higher connectivity *and* activity level have any legitimacy? Chronic, excessive metabolic demand can lead to tissue damage in many organs, and the human brain has extraordinary energy demands. Furthermore, major AD risk factors such as age, ApoE genotype, vascular damage and female gender have all been linked to an increased burden on neuronal metabolism, activity and plasticity [17]–[19]. Recently, direct evidence was presented that excessive neuronal and/or synaptic activity leads to amyloid deposition [20], [21], [22]. However, whether this relation between neuronal activity and AD pathology exists in humans, and whether hub regions are indeed the most active areas of the brain has not yet been explored. We speculated that an ‘activity dependent degeneration’ scenario, in which hub regions are preferentially affected due to high neuronal activity levels, could be a plausible disease mechanism.

To test this hypothesis, a model is required that incorporates both large-scale connectivity as well as (micro-scale) neuronal activity. The macroscopic level is needed to provide a realistic structural human brain topology, including hub regions. Topological maps are well within reach nowadays, since an increasing amount of imaging data describing the human *connectome* is becoming available [1], [23], [24]. Imposed on this structural framework, a realistic representation of network dynamics is required. For this purpose, so-called *neural mass models* (NMMs) can be employed [25]–[27]. Here, each neural mass reflects activity in a brain region by representing a large population of interconnected excitatory and inhibitory neurons, characterized by an average membrane potential and spiking density. Multiple neural masses can be coupled according to any desired structural topology (e.g. human anatomical data) to form a dynamic brain model, which can then be employed to investigate the relationship between connectivity and neuronal activity [28]–[30].

Structural (anatomical) connectivity and functional (dynamical) connectivity are strongly related, but not always in a straightforward way [5], [31]–[33]. It has been shown that macroscopic models of mammalian brain networks combined with graph theoretical analysis may explain the topology of functional networks at various time scales [34]–[36]. To simulate disease, macroscopic models and graph theory have been used to predict the structural and functional consequences of various types of lesions on brain networks [11], [12], [30]. Similarly, the gradually progressive neuronal damage of neurodegenerative processes such as AD can be modeled using this approach, and analyzed with graph theoretical tools [14], [37]–[39]. The novel aspect of the present study is that the degenerative damage is based on neuronal activity itself.

In short, by simulating neuronal dynamics on a network that is modeled on a realistic human cortical connectivity structure we explore the relation between large-scale connectivity and neuronal activity in normal and abnormal conditions. In the present study we use this approach to a) establish that cortical hub regions, *because* of their high connectivity, possess the highest intrinsic neuronal activity levels, and b) demonstrate that ‘Activity Dependent Degeneration’ (ADD), in which brain connectivity is damaged based on local neuronal activity levels, may serve as a computational model of AD that offers a potential explanation for hub vulnerability.

## Results

### Experiment 1: Relation between connectivity and activity

**Figure 1:**
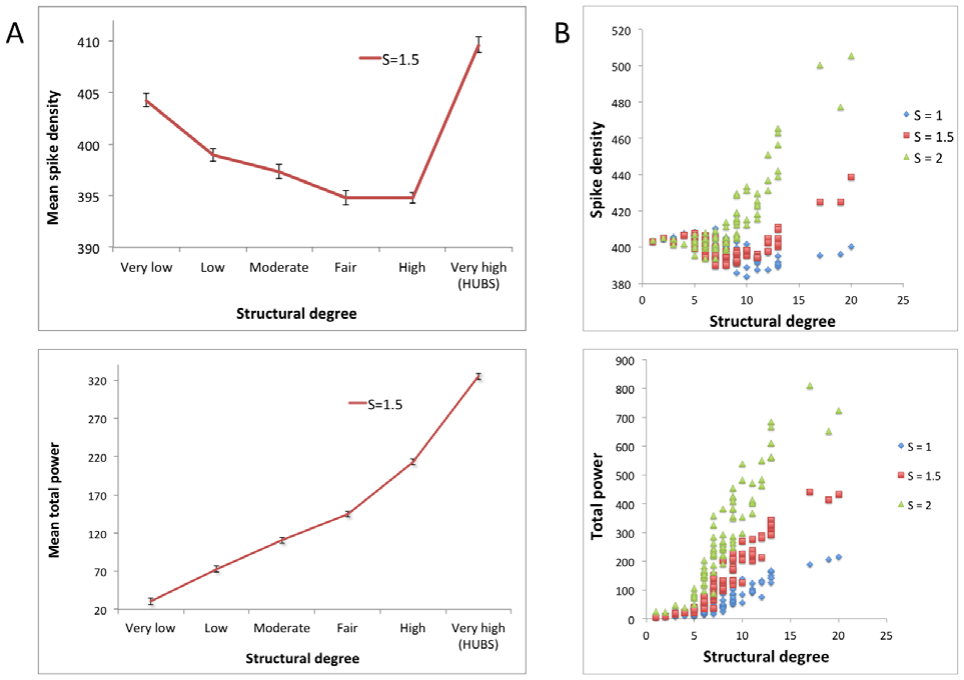
Relation between structural degree and neuronal activity. A: Six bins with ascending mean structural degrees are plotted against their average spike density and total power values. Nodes in the ‘very high’ degree bin were defined as hubs. Coupling strength (S) between neural masses was set to 1.5. Error bars indicate standard deviation within each bin. B: Similar plots as in the left panel, but for every region individually, and for three different coupling strengths S (see Text S1, section 3).

To assess whether the most highly connected cortical regions also showed the highest levels of neuronal activity, we plotted spike density and total power for all regions against the structural degree of nodes (figure 1A). The group of 13 regions with the highest (‘very high’ category in the figure) structural degree were defined as hubs; the remaining 65 regions were labeled as non-hubs. In non-hubs, spike density actually showed a weak negative relation with structural degree, but in hubs clearly higher levels were found compared to non-hubs (p<0.01). Furthermore, the total power of hubs was significantly higher than that of non-hubs (p<0.0001). Figure 1B shows the same relations, but now plotted for all regions, and for three different initial coupling strengths. When S = 1.5, the correlations between structural degree and spike density (r = 0.35) and structural degree and total power (r = 0.94) indicate that especially the link between structural degree and total power is strong. For higher coupling strengths between the NMMs (S = 2.0), a strong positive correlation between structural degree and spike density was observed as well (r = 0.86). Thus, although coupling strength has an influence on these results, overall the positive relation between structural connectivity and neuronal activity is apparent.

Since activity level might also be influenced by a nodes functional role rather than its structural connectivity status, we performed comparisons between structural and functional degree (sum of all weighted *functional* connections of a node) of all nodes for the common frequency bands (delta 0–4 Hz, theta 4–8 Hz, lower alpha 8–10 Hz, higher alpha 10–13 Hz, beta 13–30 Hz, gamma 30–45 Hz). Results of this analysis and of direct comparisons between functional degree and neuronal activity are reported in Text S1 section 1. In most bands, clear positive correlations were found, demonstrating that functional hub regions generally have high neuronal activity levels as well. Table 1 shows all 78 regions ranked by structural degree, with their functional degree, total power and spike density levels.

**Table 1:** Cortical regions; degree of connectivity and level of activity.

Cortical region	*Structural degree*	*Functional degree* *		*Spike density*	*Total power*
**Precuneus R**	20	0.034	±0.004	435	420
**Precuneus L**	19	0.034	±0.003	426	408
**Middle Occipital Gyrus L**	17	0.033	±0.004	428	447
**Superior Frontal Gyrus, medial R**	13	0.035	±0.004	395	228
**Calcarine fissure and surrounding cortex L**	13	0.035	±0.004	408	296
**Middle Temporal Gyrus L**	13	0.034	±0.004	404	275
**Superior Occipital Gyrus R**	13	0.032	±0.005	410	342
**Calcarine fissure and surrounding cortex R**	13	0.032	±0.005	412	352
**Precentral Gyrus L**	13	0.031	±0.005	403	312
**Lingual Gyrus R**	12	0.032	±0.004	403	203
**Superior Frontal Gyrus, medial L**	12	0.032	±0.005	395	226
**Middle Occipital Gyrus R**	12	0.031	±0.004	404	285
**Precentral Gyrus R**	12	0.03	±0.004	398	278
Postcentral Gyrus L	11	0.033	±0.004	396	227
Superior Frontal Gyrus, dorsal L	11	0.032	±0.005	396	242
Postcentral Gyrus R	11	0.031	±0.004	395	261
Superior Frontal Gyrus, dorsal R	11	0.03	±0.005	396	234
Superior Temporal Gyrus R	10	0.034	±0.004	397	127
Supplementary motor area R	10	0.034	±0.005	398	188
Cuneus R	10	0.034	±0.004	398	276
Superior Occipital Gyrus.L	10	0.027	±0.004	398	264
Insula L	9	0.035	±0.006	395	143
Inferior Temporal Gyrus L	9	0.033	±0.004	395	184
Lingual Gyrus L	9	0.033	±0.005	398	205
Supplementary motor area L	9	0.032	±0.005	397	131
Supramarginal Gyrus R	9	0.032	±0.005	393	175
Angular gyrus R	9	0.03	±0.006	391	200
Middle Temporal Gyrus R	9	0.03	±0.005	393	177
Fusiform Gyrus L	9	0.03	±0.005	395	167
Superior Parietal Gyrus R	9	0.029	±0.005	393	207
Middle Frontal Gyrus, R	9	0.029	±0.004	400	61
Inferior Frontal Gyrus, orbital part L	9	0.028	±0.006	398	130
Anterior Cingulate and paracingulate Gyri L	9	0.028	±0.006	395	140
Cuneus L	9	0.028	±0.004	397	86
Superior Frontal Gyrus, medial orbital R	8	0.033	±0.004	393	109
Angular gyrus L	8	0.032	±0.005	392	232
Superior Parietal Gyrus L	8	0.03	±0.004	395	163
Inferior Frontal Gyrus, opercular part.R	8	0.029	±0.006	400	64
Superior Frontal Gyrus, orbital part L	8	0.028	±0.005	395	122
Superior Temporal Gyrus L	8	0.028	±0.004	402	74
Middle Frontal Gyrus L	8	0.028	±0.005	394	123
Temporal Pole: middle temporal gyrus R	8	0.026	±0.005	396	113
Paracentral Lobule L	8	0.026	±0.005	399	101
Anterior Cingulate and paracingulate gyri R	8	0.026	±0.004	394	143
Fusiform Gyrus R	8	0.024	±0.005	394	145
Superior Frontal Gyrus, medial orbital L	7	0.032	±0.003	390	120
Median Cingulate and paracingulate gyri R	7	0.031	±0.005	402	69
Inferior Occipital Gyrus L	7	0.03	±0.005	397	127
Paracentral Lobule R	7	0.029	±0.005	403	63
Inferior Frontal Gyrus, opercular part L	7	0.028	±0.006	405	31
Supramarginal Gyrus L	7	0.028	±0.006	398	75
Gyrus Rectus L	7	0.027	±0.004	394	63
Rolandic operculum L	7	0.027	±0.005	398	110
Inferior Frontal Gyrus, triangular part L	7	0.027	±0.004	396	101
Superior Frontal Gyrus, orbital part R	7	0.026	±0.004	405	37
Inferior Parietal L	7	0.026	±0.004	402	42
Inferior Temporal Gyrus R	7	0.015	±0.003	394	109
Inferior Occipital Gyrus R	6	0.031	±0.004	409	23
Olfactory cortex R	6	0.025	±0.004	396	134
Parahippocampal Gyrus L	6	0.025	±0.006	404	47
Temporal Pole: middle temporal gyrus L	6	0.025	±0.004	402	45
Inferior Parietal R	6	0.025	±0.005	394	112
Median Cingulate and paracingulate gyri L	6	0.024	±0.004	405	43
Parahippocampal Gyrus R	6	0.023	±0.005	399	60
Rolandic operculum R	6	0.023	±0.003	410	35
Posterior cingulate Gyrus L	6	0.021	±0.003	404	43
Inferior Frontal Gyrus triangular part R	6	0.02	±0.005	404	45
Inferior Frontal Gyrus, orbital part R	5	0.024	±0.006	404	31
Insula R	5	0.021	±0.004	404	17
Temporal Pole: superior temporal gyrus L	5	0.018	±0.003	405	29
Middle Frontal Gyrus, orbital part L	5	0.017	±0.004	390	163
Posterior Cingulate Gyrus R	5	0.013	±0.002	397	225
Middle Frontal Gyrus, orbital part R	4	0.022	±0.004	406	19
Gyrus Rectus R	4	0.014	±0.002	405	29
Olfactory cortex L	4	0.013	±0.003	400	37
Temporal Pole: superior temporal gyrus R	3	0.017	±0.003	403	17
Heschl Gyrus L	2	0.012	±0.002	405	9
Heschl Gyrus R	1	0.012	±0.002	403	6

List of human cortical regions included in the model, ranked in order of descending structural degree. Regions printed in bold were classified as hub regions.

* Functional degree is based on broadband (0.5–45 Hz) functional connectivity.

S (coupling strength) was set at 1.5; different values of S produced different absolute values but no changes in functional degree rank. T (time delay) was kept constant at 0.002 s for all experiments (see Text S1, section 2). Averaged values and standard deviations over 20 runs of the NMM.

### Experiment 2: Activity Dependent Degeneration (ADD)

#### Effect of ADD on structural network integrity

**Figure 2:**
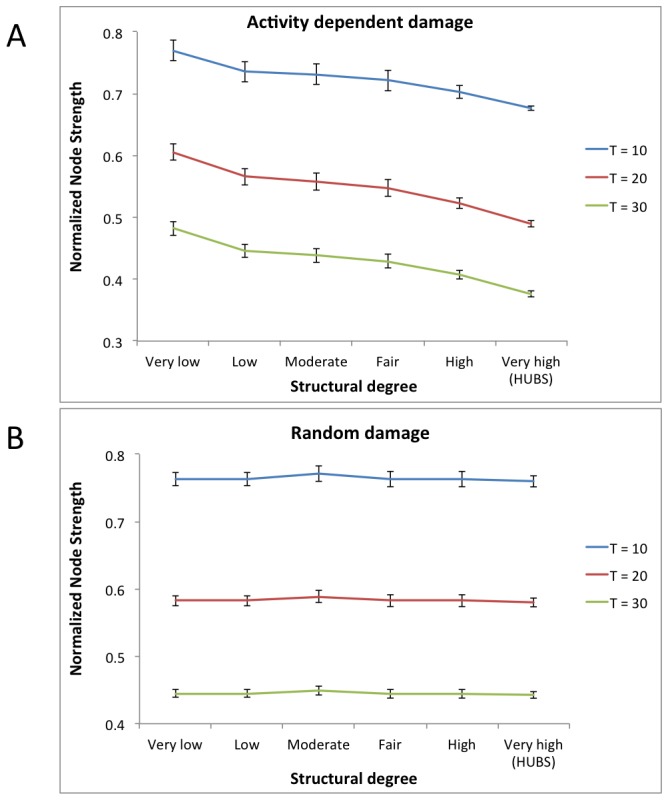
Effect of ADD on structural degree. A: All cortical regions binned according to initial structural degree from low to high values, and their average normalized node strengths at different stages of activity dependent degeneration (ADD). T = time. Error bars indicate standard error of the mean. B: All cortical regions binned according to initial structural degree from low to high values, and their average normalized node strengths at different stages of random degeneration (RD). T = time. Error bars indicate standard error of the mean.

Since, according to our hypothesis, ADD lowers connectivity based on activity level, it was expected to disrupt both structural and functional networks. First we investigated the effect of ADD on the structural network, and whether it had different effects on hubs versus non-hub regions. In ADD, every time-unit represents a small amount of damage to the system, so as to simulate gradual, cumulative degeneration. However, the amount of real, absolute time that is required for these successive steps is not known. Time as presented in these figures should therefore not be interpreted as days or years, but as arbitrary units of undetermined length. Figure 2A shows the decrease of the structural connectivity for three time points in all regions. The normalized node strength, which is the ratio of the node strength after ADD over the original node strength, is plotted for different time points. At baseline (T = 0, not shown) normalized node strength is 1 by definition. Over time node strength decreases, and, as hypothesized, particularly in hub nodes, illustrated by the declining slope of the lines. The difference in normalized node strength between hubs and non-hubs is highly significant for all time points shown (p<0.001). On the contrary, in the random degeneration (RD) model, there was no difference between hubs and non-hubs in normalized node strength over time (see figure 2B).

#### Effect of ADD on neuronal activity

**Figure 3:**
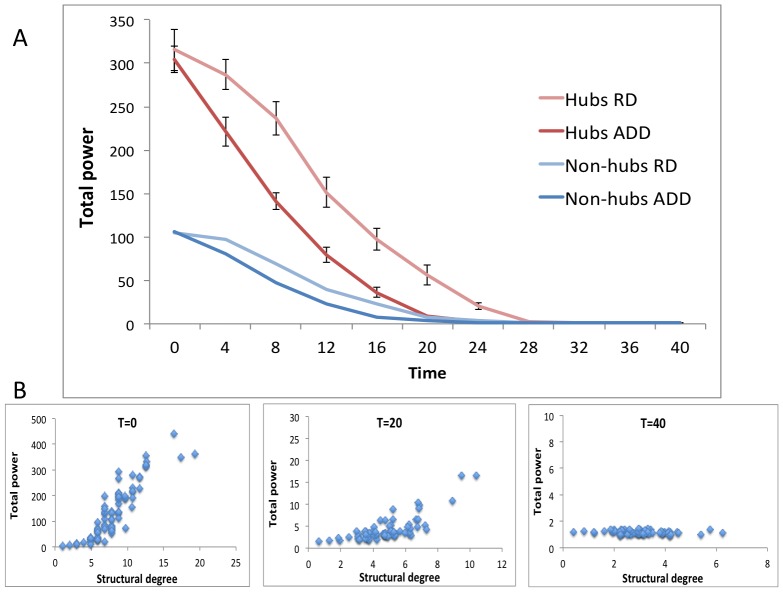
Effect of ADD on total power. A: Average total power of hub and non-hub regions plotted over time, for both the ADD and RD procedure. Error bars indicate standard error of the mean. B: Correlation between structural degree and total power for all regions at different time points during ADD.

Next, we studied the effect of ADD on network dynamics. When visually inspecting the model-generated data it was apparent that there were notable changes in oscillation amplitude over time. The power spectrum of hub regions initially showed much higher alpha power than in non-hub areas, and a surprising slightly lower alpha peak frequency (see Text S1 section 4). As expected, total power decreased over time (see figure 3A). Hubs started at a higher mean power level (p<0.0001), but declined more rapidly than non-hubs, reaching bottom levels at approximately the same moment. Loss of total power in the ADD model was stronger than in RD, especially in hubs; for all time points (except T = 0) hub power under ADD was significantly lower than under the RD regime (p<0.01). The initial positive relation between structural degree and total power disappeared accordingly (see figure 3B).

**Figure 4:**
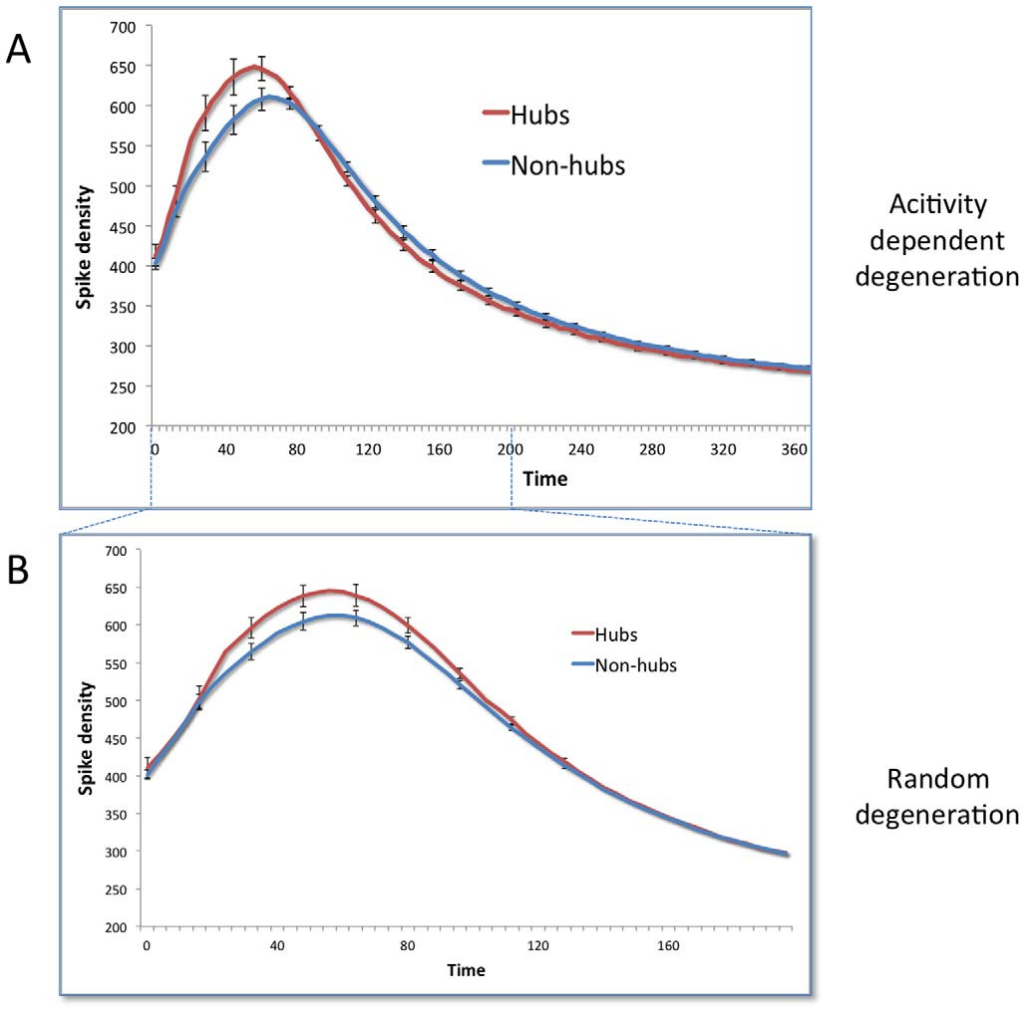
Effect of ADD on spike density. A: Average level of spike density during ADD is plotted for hubs and non-hubs. Error bars indicate standard deviations. B: Average level of spike density during RD is plotted for hubs and non-hubs. Error bars indicate standard deviations.

We subsequently performed a similar analysis for spike density changes over time due to ADD and RD (see figure 4A and 4B). At T = 0, the spike density in hubs was higher than in non-hubs (p = 0.01). In the early stage, we found an unexpected rise of spike density in both ADD and RD, which was stronger in hubs (maximum spike density increase was larger, p<0.0001). However, the maximum spike density in hubs under ADD was reached significantly earlier than in non-hubs (average T = 52 versus T = 60, p<0.0001), while peaks were reached at similar times under RD.

#### Effect of ADD on functional network topology

**Figure 5:**
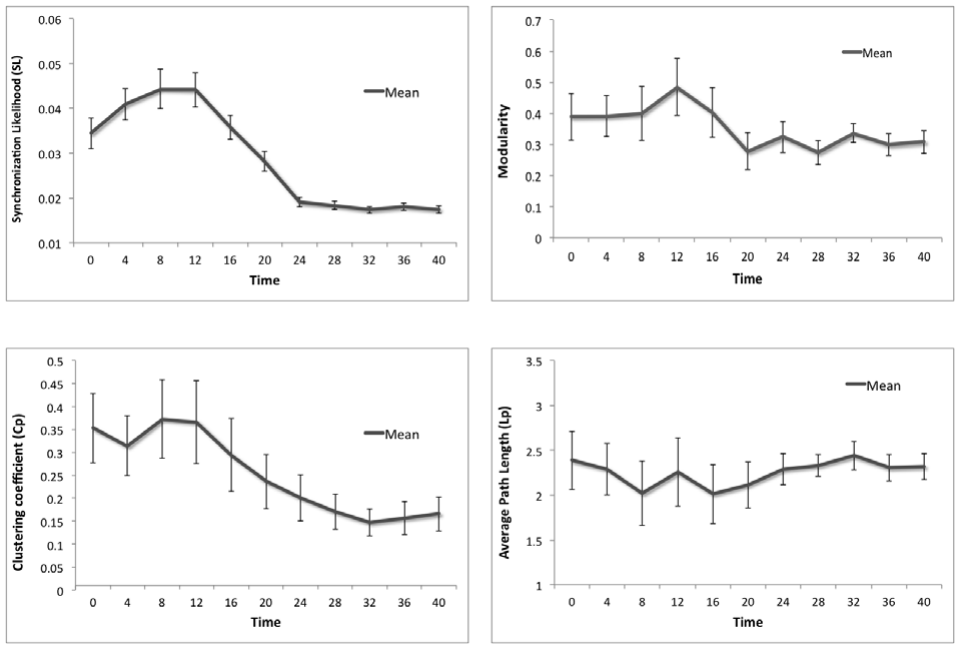
Effect of ADD on functional connectivity and network topology. Mean levels of synchronization likelihood, modularity, clustering coefficient and path length during ADD are plotted for hubs and non-hubs. Error bars indicate standard deviations.

Since we expected ADD to affect functional network topology as well, we examined changes over time in the synchronization likelihood, as well as basic graph measures like average clustering coefficient, characteristic path length, and modularity. Since data generated by the NMM is most reliable in the alpha band, and AD-related functional network changes have most consistently been found in the lower alpha band, we report just the results of this representative band in figure 5. Like spike density, functional connectivity strength first increased before a rapid breakdown occurred, which reached bottom level at around the same time point as total power (described above). The average clustering coefficient decreased, while the characteristic path length fluctuated around the same level through the ADD process (although hubs and non-hubs showed different behavior during the first phase, see figure 5). The ratio between these two measures became smaller, indicating that the balance between global and local connectivity and thus the small-world network topology was disturbed and had become more random. Global modular organization, as expressed by Newman's index, decreased before reaching a stable, lower level.

## Discussion

In this study we used a computational model with 78 dynamic neural masses coupled according to realistic human cortical topology to investigate the relation between connectivity and neuronal activity. We find that cortical hub regions have the highest level of intrinsic activity, and that the minimal assumption of higher local neuronal activity leading to more severe neurodegeneration can predict a range of AD hallmarks observed in patient data such as oscillatory slowing, a subsequent increase and breakdown of functional connectivity, and a loss of functional network integrity. These results suggest an ‘activity dependent degeneration’ (ADD) hypothesis of AD, and below we will discuss our findings and possible consequences in greater detail.

### Hub status and activity level

Our first aim was to find out whether the level of activity in a region is related to its degree of structural connectivity. An expected positive correlation was indeed found in repeated experiments across all degrees of connectivity (see figures 1, 3, and 4): structural hub regions possess the highest average power and spike densities. As can be judged from figure 1, an exception is the relation between structural connectivity and spike density for low values of NMM coupling (S). This result indicates that the relation between connectivity and activity might be more complex than we expected. Nevertheless, similar analysis performed using functional connectivity results (see figure S1) led to clear positive correlations in the large majority of cases. It should further be noted that there is no unique definition of hub status, and in this experiment (and the rest of the study) we adhered to the pragmatic choice of taking a selection of nodes (n = 13) with the highest structural degree. However, since connectivity and activity are clearly positively related in regions with higher structural degrees, we do not believe that a different hub definition would have led to a different interpretation.

Still, although high neuronal activity in hub regions was a solid finding that might have been expected intuitively, it should ultimately be verified in experimental data. As can be judged from table 1, many Default Mode Network (DMN)-related regions possess a high degree of connectivity *and* activity. The well-documented high resting-state activity level of the DMN is therefore in line with our findings [5]; however, instead of being attributed to ongoing cognitive processing or mental phenomena like introspection, high resting-state activity in the DMN might actually be (partially) explained by the underlying degree of structural and functional connectivity

### Activity Dependent Degeneration (ADD)

Based on the findings in our first experiment, we expected that ADD would probably preferentially target hub regions, since they possessed the highest level of activity. Analyses of both structural and functional connectivity changes due to ADD seem to be in agreement with this expectation (see figures 2–5). Furthermore, total (or absolute) power decreases rapidly, largely accounted for by weakening of hub regions, and the initial correlation between degree and power is lost (figure 3). Thus, large-scale brain connectivity loses its efficient ‘hub’ topology in ADD, like in AD.

**Figure 6:**
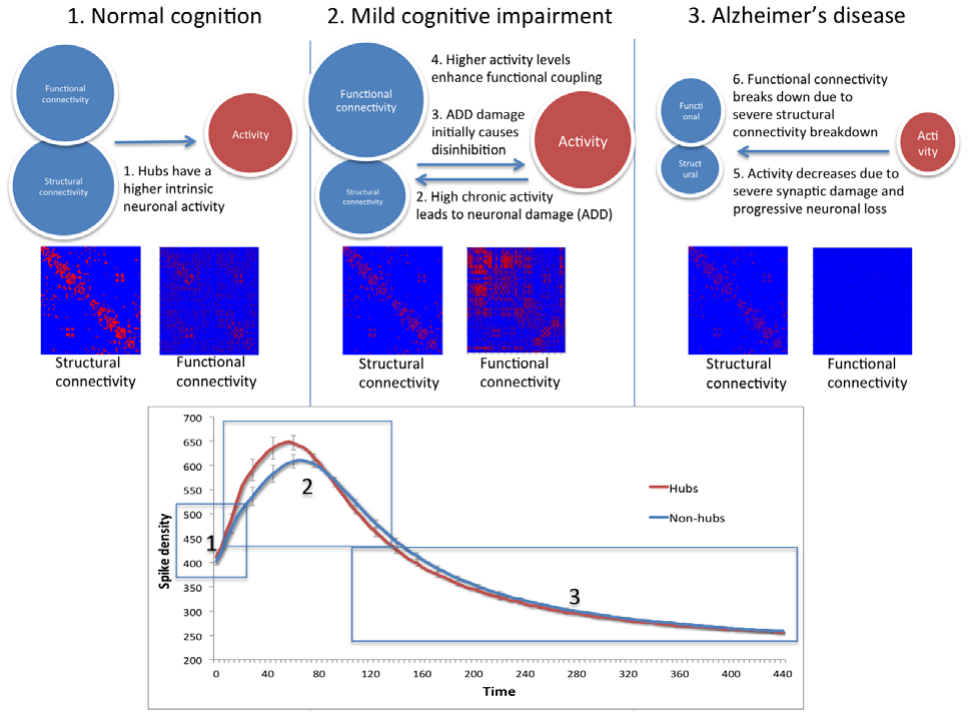
The relation between connectivity and activity at different stages of ADD. The proposed relation between connectivity and activity is summarized for three different stages of ADD. Structural hubs have a higher baseline intrinsic activity, making them most susceptible to ADD. The second phase might represent the ‘Mild Cognitive Impairment’ (MCI) stage; structural connectivity declines steadily, but functional connectivity, power and spike density initially increase, leading to a pathologic spiral of increasing activity and metabolic burden in progressively weaker neurons. In the third “AD” phase, the damaged neurons and decreasing structural connectivity can no longer support the high demands, and the network collapses.

Surprisingly, the steady loss of power is accompanied by an initial rise of spike density on average (see figure 4), before a final oscillatory slowing sets in. This effect is stronger in hubs; spike density rises more quickly, reaches its peak rate sooner, and seems to slow down more rapidly. One explanation for the increase in spike density observed in our results is neuronal disinhibition. In fundamental neuroscience disinhibition is a well-known phenomenon and it is widely accepted that inhibitory interneurons have a large influence on oscillatory behavior [40]. Besides damaging excitatory connections, ADD impairs connectivity to and from inhibitory neurons within the neural masses, and the resulting loss of inhibition seems to be a dominant influence on spike density in the first stage. This then leads to a vicious spiral of increasing activity, more activity-dependent damage, etc. until the weakening network can no longer support an increase in spike density (the inter-mass excitatory coupling weakens substantially, which leads to breakdown of the system, see also figure 6). The eventual spike density decrease due to ADD resembles the oscillatory slowing known from AD neurophysiologic literature [41], [42].

Several authors have argued for a prominent role of neuronal disinhibition in AD pathophysiology: for example, Gleichmann et al. propose a process they call ‘homeostatic disinhibition’, which is based on a different underlying mechanism but might explain the higher prevalence of epilepsy that is seen in AD, reduced gamma band activity, and, interestingly, the increase in neuronal activity as measured by fMRI [43]. Schmitt argues that AD is accompanied by a loss of inhibition that leads to alterations in calcium homeostasis and excitotoxicity, respectively [44]. Olney et al. hypothesize that a disinhibition syndrome caused by hypoactive NMDA receptors triggers excitotoxic activity and widespread neurodegeneration [45]. Palop & Mucke suggest that amyloid itself causes dysfunction of inhibitory interneurons causing an increase in neuronal activity [46], [47], possibly also accounting for the higher prevalence of epileptic activity in AD [48]. Kapogiannis & Mattson review reports that in aging excitatory imbalance is due to a decrease in GABA-ergic signaling, and that this mechanism is exacerbated in AD [19].

An early but transient rise was also found in functional connectivity results (see figure 5), and interestingly, this is in line with experimental data of Mild Cognitive Impairment (MCI) patients, where increased functional connectivity levels are often interpreted as a compensatory mechanism [49]–[52]. However, this increase of functional connectivity has not been directly related to cognitive improvement, and according to our model, it might well be a part of the degeneration process itself.

Finally, the ADD induced changes in functional network topology, such as the weakening of small-world structure and modularity (see figure 5), are in line with recent findings in resting-state EEG and MEG studies in AD [14], [39], [53]–[55]. In recent years, brain disconnectivity and disturbed network topology has been observed in an increasing number of disorders (for example schizophrenia, multiple sclerosis, brain tumor, autism, epilepsy) [56]–[59]. It is conceivable that different disease mechanisms and types of network damage (for example extensive non-hub network damage) could lead to a similar situation of hub overload and decay. Computational models like the one described here could be employed to investigate various underlying pathologies and to examine the differences between them. Several recent studies support the notion that node properties such as degree and centrality may play a crucial role in the pathophysiology of degenerative brain disease [60]–[62].

### Alzheimer's disease: consequence of excessive hub activity?

The results of this study suggest that hub regions are vulnerable due to their intrinsically high activity level. The assumption of activity dependent degeneration leads to hub vulnerability along with many neurophysiologic features of AD (i.e. as found in quantitative EEG and MEG literature). A recently conducted large fMRI study demonstrated that highly connected cortical regions like the precuneus are even stronger hubs in females than in males: could this perhaps explain the higher levels of early amyloid deposition ánd the higher prevalence of AD in women [63], [64]? The computational model used in this study offers a possible mechanism by which excessive neuronal activity in hubs might lead to the observed macro-scale disruption of brain connectivity and dynamics in AD.

**Figure 7:**
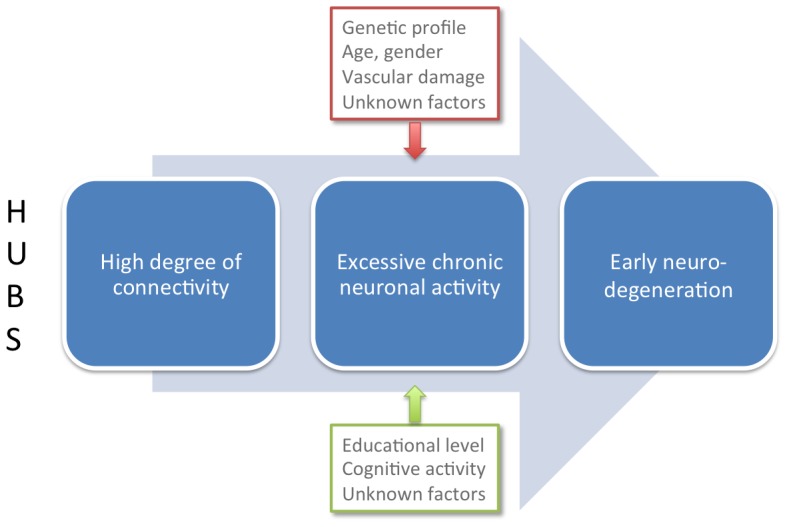
The role of excessive neuronal activity in Alzheimer's disease. Excessive neuronal activity might be a common pathway through which many of the known risk factors enlarge the chance to develop Alzheimer pathology. Hub regions are most likely to display activity-dependent pathology, since they have the highest intrinsic neuronal activity (which is further amplified in the initial phase of ADD).

In addition to the presumed role of disinhibition mentioned in the previous paragraph, a prominent role of excessive neuronal activity in AD pathogenesis has been suggested before: several studies have demonstrated a direct link between neuronal activity and the development of amyloid plaques in transgenic mice [20], [21], [22]. Regions that are most active during resting-state show the most outspoken AD-related pathology [4], [5], [13]. Excessive hippocampal activity is related to cortical thinning in non-demented elderly persons, is present in MCI patients, and is related to neurodegeneration in AD [49], [65], [66]. Finally, known risk factors for AD such as genetic profile, age, vascular damage, or common comorbidities like sleep disorders and epilepsy, all predispose to excessive activity and a subsequent burden on metabolism and plasticity [17], [18], [66]–[68]. On the other hand, protective factors like high level of education and sustained cognitive activity might relieve the burden on hub regions due to frequent activation of task-related circuits, and accompanying DMN *de*activation. Summarizing, vulnerability of cortical hub regions due to their high activity levels may be aggravated or alleviated by the presence of one or more predisposing or protective factors, respectively (see figure 7).

This line of reasoning implies that changes in brain activity and connectivity are already involved in the very early stages of AD pathology. In this regard, it is interesting to note that an increasing number of studies show that changes in activity and functional connectivity can be detected before cognitive complaints arise or pathological levels of amyloid are detected with PET and CSF analysis [18], [69]–[73].

Although activity dependent degeneration is quite different from amyloid-induced damage, they need not be mutually exclusive: chronic, excessive activity might lead to amyloid deposition, which in turn causes aberrant activity and neuronal damage: a pathological cycle with different stages (see also figure 6). Relatively small increases of extracellular amyloid-beta can increase neuronal activity, especially in neurons with low activity, whereas higher levels cause synaptic depression [74], [75]. Palop and Mucke emphasize the role of inhibitory interneuron dysfunction, leading to hypersynchronization [47]. In conclusion, although these studies provide compelling evidence for a prominent role of neuronal activity, our predictions that hub regions might form the weakest links in AD pathogenesis should be tested in further studies.

### Modeling Alzheimer's Disease

Several recent studies use similar computational modeling approaches to examine AD related neurophysiological phenomena: Bhattacharya et al. focus on thalamo-cortico-thalamic circuitry and its relation with alpha band power in AD [38]. By varying the synaptic strengths in the thalamic module of the model they find that especially the connectivity of synaptic inhibitory neurons in the thalamus has a large influence on alpha power and frequency. Pons et al. use a neural mass model and human EEG data to investigate the influence of structural pathways on functional connectivity in the aging brain and pre-clinical stages of AD [37]. Findings in line with our present results are the higher functional connectivity values in MCI and the relation between structural and functional connectivity. An increase in functional connectivity and network randomness during a memory task was found by Buldú et al. in a MEG study of MCI patients [76]. Interestingly, the authors also provide a network degeneration model which might explain these observations. The combination of neural mass modeling and graph theory was used in a recent study from our group [36]. This study explores the manifestation of modularity in developing networks and investigates the effect of more acute lesions on network dynamics. The gradual recovery of functional network characteristics that was observed after lesions raises the question whether and to what extent similar mechanisms play a role in neurodegenerative damage; this should be subject of further study. To describe functional network modularity, the same algorithm and heuristic was used as in the present study. The computational models used in these studies provide a framework to address different questions and hypotheses concerning brain disease, e.g. different functional lesions. A novel aspect of the approach in the present study is that a single hypothesis (ADD) is proposed as main pathophysiological mechanism of AD. Comparison to a ‘random degeneration’ (RD) model provides further support for the ADD hypothesis, but does not rule out the possibility that other plausible degenerative models exist.

### Methodological issues

Various methodological choices might have affected our results, and should be taken into account when interpreting them. First, although the DTI-derived connectivity matrix that served as the basis of our model is in our opinion a solid overall large-scale representation of human cortical connectivity, it was based on data of healthy young adults [24]. Since AD mainly affects the aging population, and since it has been shown that structural connectivity is altered during aging [77], results might have been different if structural connectivity data of older subjects had been implemented. However, the major hub regions seem largely independent of age, justifying our approach that mainly focuses on hub versus non-hub differences. Furthermore, we now know that AD affects many people below the age of 65, and that AD pathology is presumably already present for decades before initial symptoms appear. In a similar way we expect that individual variability in structural connectivity will not have had a major influence on our present approach, since major hub regions appear to be consistent across studies [3], [64]. Although the computational model used here could be refined in many ways, e.g. by implementing a larger number of regions, assigning different weights to structural connections, using DSI-derived data, correcting for spatial scale and/or DTI biases, or by using more elaborate and detailed graph analysis, we believe that this would not have affected our main outcome dramatically, since comparing characteristics of hub and non-hub cortical regions does not necessarily require a high level of detail. By keeping the model and hypotheses as simple as possible, it might be easier to discover or test underlying basic principles and mechanisms of degeneration.

The main motivation to use an NMM network of this size was the observation that topographical maps and atlases of the human cerebral cortex of this order of magnitude are quite common in macroscopic structural and functional connectivity studies (for an overview, please refer to [39], [56]–[58]. Also, since EEG and MEG studies have comparable network sizes (21–300 sensors), this is a fairly realistic spatial resolution for NMM-generated dynamics. Two relevant references are recent computational modeling studies by Deco et al. [27] and Pons et al. [37].

Varying the structural coupling strength S in our neural mass model can lead to different results, and therefore we have reported its influence on our outcomes. Similarly, the arbitrary ‘loss’-rate parameter of the ADD function will affect the speed of the degeneration process. However, since we were mainly concerned with a topological ‘hub versus non-hub’ comparison, the absolute rate of degeneration was of minor importance for this study. Moreover, loss-rates were equally applied to *all* connections; network *distribution* was not selectively influenced by these parameters.

### Future directions

Observations from this study that could be explored further include ADD-induced changes in structural network topology, the relation between spike density and anatomical region, and the lower alpha peak frequency in hub regions (see Text S1 section 4). Predictions from our model, especially the close link between local neuronal activity and large-scale connectivity should be verified in longitudinal clinical studies, preferably of normal aging as well as patients with subjective memory complaints (SMC), Mild cognitive impairment (MCI) and AD. To assess structural and functional connectivity as well as large-scale neuronal activity, a combination of DTI and MEG might be the most appropriate method. Source space analysis of MEG data may help to develop topologically accurate neural mass models. On a fundamental level, the relation between neuronal connectivity, activity and pathology should be further explored in animal models. Interestingly, the relation between regional activity and large-scale functional connectivity has recently also addressed with respect to schizophrenia [78], [79]. In both studies it is argued that more knowledge of this relation is essential for understanding mechanisms of altered functional connectivity, and this is very much in line with the main message of this study. Different disease conditions may have specific causes or patterns in which this relationship is harmed, but at the same time universal principles may apply that can help us gain more insight in a range of neuropsychiatric disorders.

### Conclusion

In this study we used a neural mass model with DTI-based human topology to demonstrate that brain hub regions possess the highest levels of neuronal activity. Moreover, ‘Activity dependent degeneration’ (ADD), a damage model motivated by this observation, generates many AD-related neurophysiologic features such as oscillatory slowing, disruption of functional network topology and hub vulnerability. Early-stage, transient rises of firing rate and functional connectivity in ADD matches observations in pre-clinical AD patients, suggesting that this chain of events is not compensatory, but pathological. Overall, the results of this study favor a central role of neuronal activity and connectivity in the development of Alzheimer's disease.

## Materials and Methods

**Figure 8:**
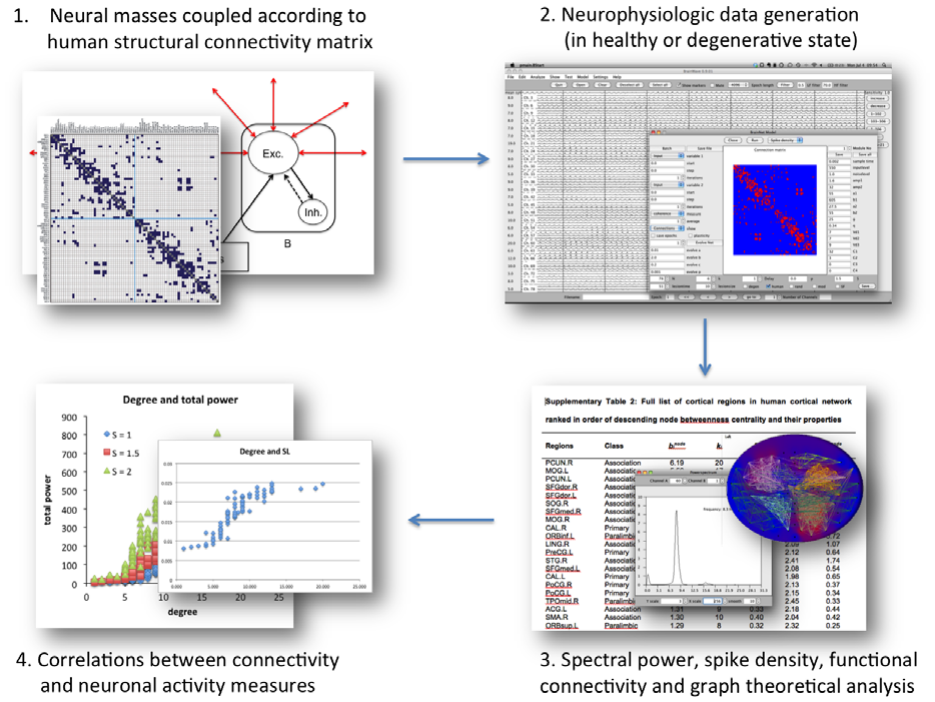
Outline of the consecutive steps in the experimental procedure. Multi-step procedure from the simulation of realistic human neurophysiological activity to analyzing and correlating connectivity and activity results.

In this study we simulated neurophysiologic activity of 78 Neural Mass Models embedded in a realistic structural cortical network topology to evaluate hypotheses about the relation between (structural and functional) connectivity and neuronal activity. The output of this model provides information about the neuronal activity level in the form of average voltage and spike density per region, and generates EEG-like data that can be subjected to further spectral, functional connectivity and graph theoretical analysis. Hypotheses about brain pathophysiology can be tested by artificially damaging structural or dynamical properties of the brain model. The outline of the analysis procedure employed in this study is depicted in figure 8.

### Network dynamics: Description of the Neural Mass Model

We used a model of interconnected neural masses, where each neural mass represents a large population of connected excitatory and inhibitory neurons generating an EEG or MEG like signal. The model was recently employed in two other graph theoretical studies [30], [36]. The basic unit of the model is a neural mass model (NMM) of the alpha rhythm [26], [80], [81]. This model considers the average activity in relatively large groups of interacting excitatory and inhibitory neurons. Spatial effects (i.e. distance) are ignored in this model; brain topology is introduced later by coupling several NMMs together. The average membrane potential and spike density of the excitatory neurons of each of the NMMs separately were the multichannel output related to neuronal activity that was subject to further analysis. All neural mass model parameters and functions are summarized and explained in Text S1, section 3 (see also figure S4 and table S1).

### Network wiring: Realistic human network

A diffusion tensor imaging (DTI) based study by Gong et al. published in 2009 that focused on large-scale structural connectivity of the human cortex resulted in a connectivity matrix of 78 cortical regions [24], [82]. The connectivity matrix was implemented in our model software, and used as topological framework for the 78 coupled NMMs. Coupling between two NMMs, if present, was always reciprocal, and excitatory. Note that at the start of the simulation, the coupling strength between all NMM pairs (S) was identical, and the only difference between the cortical regions (or NMMs) was their degree of connectivity to other neural masses (cortical regions). Since the coupling strength S was an arbitrarily chosen parameter, repeated analyses were performed with different values of this variable (see for example figure 3).

### Activity Dependent Degeneration (ADD)

For the present study the model was extended to be able to deal with activity dependent evolution of connection strength between multiple coupled NMMs. Activity dependent degeneration (ADD) was realized by lowering the ‘synaptic’ coupling strength as a function of the spike density of the main excitatory neurons. For each neural mass the spike density of the main excitatory population is stored in a memory buffer that contains the firing rates of the last 20 steps in the model. Step size depends on the sample frequency. At each new iteration, the highest spike density value of the last 20 sample steps is determined and designated as maxAct. From maxAct a loss is determined according to the following relation:
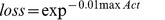
(1)Since maxAct is non-negative, loss will be a number between 0 and 1. Next, the coupling values C1 (connections between main excitatory population and inhibitory population), C2 (connections between inhibitory population and main excitatory population), Pt (thalamic input to main excitatory population) and S (structural coupling strength between neural masses) are all multiplied by loss to obtain their new lower values. To assess the specificity of ADD, results were compared with a random degeneration (RD) model in which the *maxAct* variable was discarded, so damage was equally applied to all regions, regardless of their level of activity. The effects of ADD and RD were measured by changes in total power (local average membrane potential) and spike density, and these two measures were used as representations of neuronal activity in further analyses. Note that the time scale of the data generated by the model is equal to normal brain activity and EEG/MEG data, but that the ADD and RD procedures have a more abstract time scale. The exact duration of the degenerative procedures was not considered relevant to our present focus on the relation between connectivity and activity, but could be considered to reflect a length that is representative of a neurodegenerative process, spanning years to decades (see figures 3–5). The computational model was programmed in Java and implemented in the in-house developed program BrainWave (v0.9.04), written by C.J. Stam (latest version available for download at http://home.kpn.nl/stam7883/brainwave.html).

### Spectral analysis

Since spectral analysis is a common neurophysiological procedure that provides clinically relevant information in neurodegenerative dementia, we included this in our experiments. Fast Fourier transformation of the EEG-like oscillatory output signal was used to calculate for all separate regions the total power (absolute broadband power, 0–70 Hz) as well as the absolute power in the commonly used frequency bands delta (0.5–4 Hz), theta (4–8 Hz), lower alpha (8–10 Hz), higher alpha (10–13 Hz), beta (13–30 Hz) and gamma (30–45 Hz).

### Functional connectivity analysis

To quantify large-scale synchronization as a measure of interaction between different cortical areas, we used the Synchronization likelihood (SL), which is sensitive to both linear and non-linear coupling [83], [84]. SL was calculated for all frequency bands, and the matrix containing all pairwise SL values served as the basis for all further graph theoretical analyses of functional network characteristics.

### Graph theoretical analysis

Graph theoretical properties of the structural DTI network that were relevant for our hub study such as node degree, betweenness centrality, and local path length were published in the original article by Gong et al [24]. One new measure we introduced was the ‘normalized node strength’, which is the ratio of the structural degree of a node after activity dependent damage over its original degree. This measure was used to track structural connectivity loss and to compare the loss of degree in hubs and non-hubs. For functional network analysis, connectivity matrices were subjected to topographical analysis. The functional degree of a node is defined as the sum of all its link weights [85]. Averaging the functional degree over all nodes gives the overall functional degree of a network. To match the functional network to the given structural network (minimizing effects of graph size and density), we constructed a binary, unweighted matrix that was obtained after using a threshold that resulted in a network with an average degree of 8, close to that of the structural network (which was 8.1). All graph theoretical measures used in this study are summarized in table 2, for more exact definitions please refer to [14], [85]. For functional modularity analysis, we used Newman's modularity metric combined with a simulated annealing process (previously described in [55], [86]).

**Table 2:** Graph theoretical definitions.

Measure		Description
Degree	k	Number of connections of a node. Average for all nodes in a network produces the average degree K.
Node strength (or weighted degree)	k^w^	Sum of all connection weights of a node.
Clustering coefficient	Cp	Number of directly connected neighbors of a node divided by the maximally possible number of interconnected neighbors. The mean of this value for all nodes gives the average clustering coefficient; a measure of local integration.
Path length	Lp	Shortest number of steps from one node to another. Average over all possible shortest paths is the characteristic path length of a network; a measure of global integration.
Gamma	γ	Normalized average clustering coefficient, obtained by dividing Cp by the average Cp of a set randomized networks of the same size and density.
Lambda	λ	Normalized characteristic path length, obtained by dividing Lp by the characteristic Lp of a set randomized networks of the same size and density.
Modularity	Q	Expresses the strength of the modular character of a network.

Glossary of graph theoretical measures used in this study. For exact definitions, please refer to [14], [85].

### Statistical analysis

For the baseline, pre-ADD analysis in experiment 1 and 2, the data-generating procedure using the model was repeated twenty times to obtain a representative amount of data. On each run the subsequent spectral, functional connectivity and graph theoretical analysis was performed, and then all results of these twenty runs were averaged prior to further statistical analysis. Regional results were visualized using 6 bins ascending in structural degree, each containing 13 regions. All 13 regions in the bin with the highest mean degree were classified as hubs. Standard deviations of bins are displayed as error bars. For bivariate correlations Pearson's test was used.

## Supporting Information

Figure S1 Correlation between functional degree and total power in all frequency bands.
(TIF)

Figure S2 Relation between structural and functional connectivity. Left panel: matrix of the structural connections between all 78 cortical regions, adapted from Gong et al. [24]. Red squares indicate the presence of a connection. Since all connections are bidirectional, the matrix is symmetrical over its diagonal axis. Right panel: matrix of functional connections acquired using the synchronization likelihood (SL) as coupling measure (broadband frequency range: 0.5–45 Hz), and thresholding all pairwise SL values to obtain a graph with the same average degree (K = 8) as the structural connectivity matrix to the left.
(TIFF)

Figure S3 Relation between structural and functional degree in all frequency bands. Error bars depict standard deviations within each bin after 20 simulated runs.
(TIF)

Figure S4 Specifications of the neural mass model. A: Schematic presentation of single neural mass model. The upper rectangle represents a mass of excitatory neurons, the lower rectangle a mass of inhibitory neurons. The state of each mass is modeled by an average membrane potential [Ve(t) and Vi(t)] and a pulse density [E(t) and I(t)]. Membrane potentials are converted to pulse densities by sigmoid functions S1[x] and S2[x]. Pulse densities are converted to membrane potentials by impulse responses he(t) and hi(t). C1 and C2 are coupling strengths between the two populations. P(t) and Ej(t) are pulse densities coming from thalamic sources or other cortical areas respectively. B: Coupling of two neural mass models. Two masses are coupled via excitatory connections. These are characterized by a fixed delay T and a strength g. C: Essential functions of the model. The upper left panel shows the excitatory [he(t)] and inhibitory [hi(t)] impulse responses of Eq. 1. The upper right shows the sigmoid function relating average membrane potential to spike density (Eq. 2).
(TIF)

Figure S5 Power spectrum of hubs. Power spectrum of a hub region (precuneus) in black, and a non-hub region in blue. Note the difference in power, but also the lower alpha peak of the hub region.
(TIFF)

Figure S6 Alpha peak frequency in hubs. The alpha peak frequency of all cortical regions plotted against their structural degree. A negative correlation can be observed (r = −0.53). Hubs (the 13 regions with highest structural degree) have a significantly lower alpha peak (p<0.001) compared to non-hubs.
(TIFF)

Table S1 Overview of model parameters. The final model consisted of 78 of the NMMs as described above, which were coupled together based on the structural DTI network results from Gong et al. [24]. Coupling between two NMMs, if present, was always reciprocal, and excitatory. The output E(t) of the main excitatory neurons of one NMM was used as the input for the impulse response he(t) of the excitatory neurons of the second NMM; the output E(t) of the second module was coupled to the impulse response he(t) of the excitatory neurons of the first NMM. Following Ursino et al. [87] we used a time delay (T×sample time, with n an integer, 0<T<21) and a gain factor. In the present study, n and gain were set to 1 for all connections. A schematic illustration of the coupling between two NMMs is shown in Figure 1B. For the present study the model was extended in order to be able to deal with activity dependent degeneration of connection strength between multiple coupled NMMs. Coupling strength between neural masses was initially set at the same level for all connections; different levels were tested (S = 1, S = 1.5, S = 2; see figure 3).
(TIF)

Text S1 Supporting information. 1. Relation between functional degree and total power. 2. Relation between structural and functional degree. 3. Network dynamics: the neural mass model. 4. Relation between structural degree and alpha power peak frequency.
(DOC)
