# Supplementary figures and images for: Activity Dependent Degeneration Explains Hub Vulnerability in Alzheimer's Disease
Source: PLoS Comput Biol. 2012 Aug 16;8(8):e1002582. doi: 10.1371/journal.pcbi.1002582 (PMC3420961; doi:10.1371/journal.pcbi.1002582)

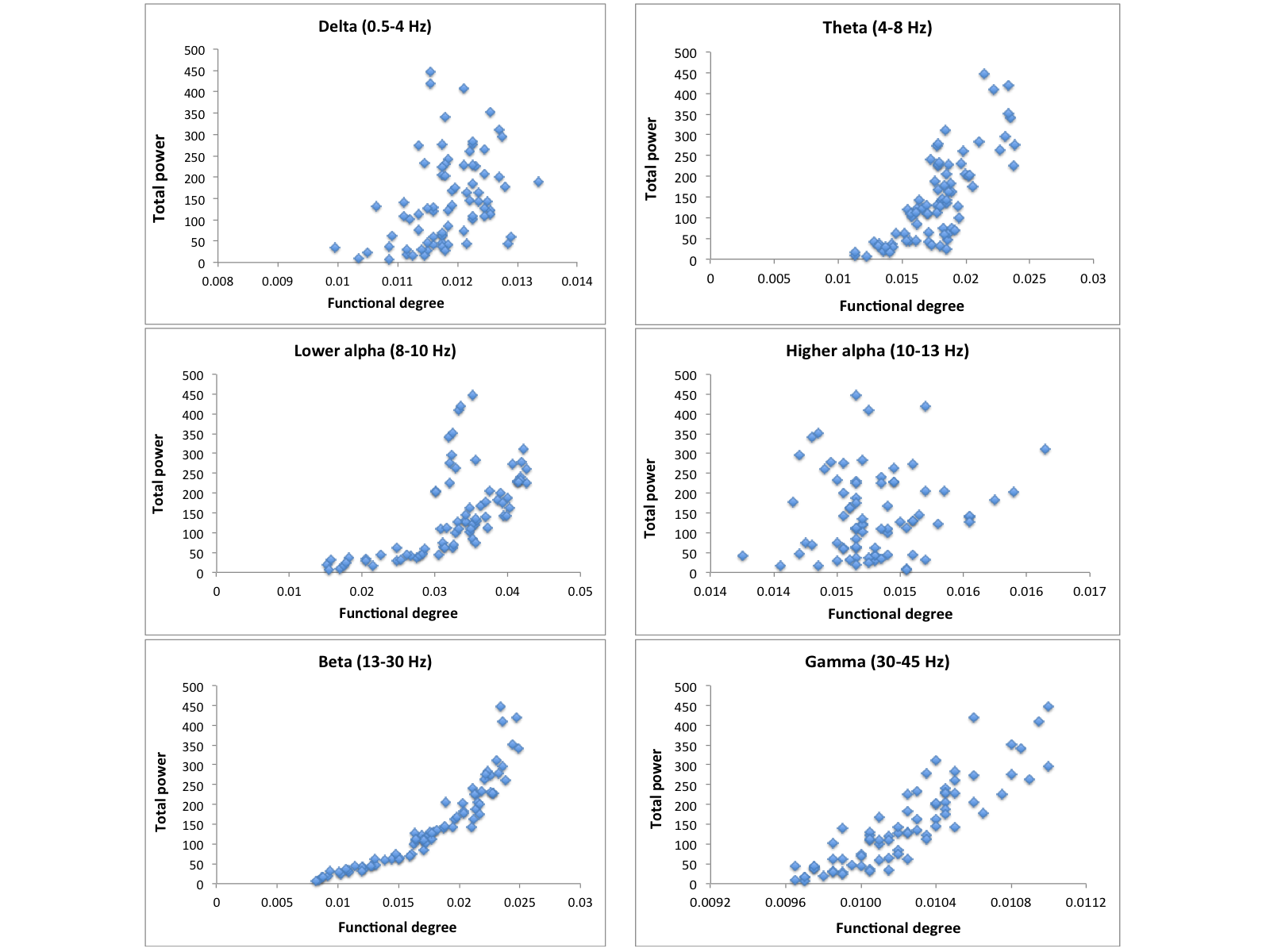

Supplement: Figure S1 — Correlation between functional degree and total power in all frequency bands. (TIF) [file pcbi.1002582.s001.tif]

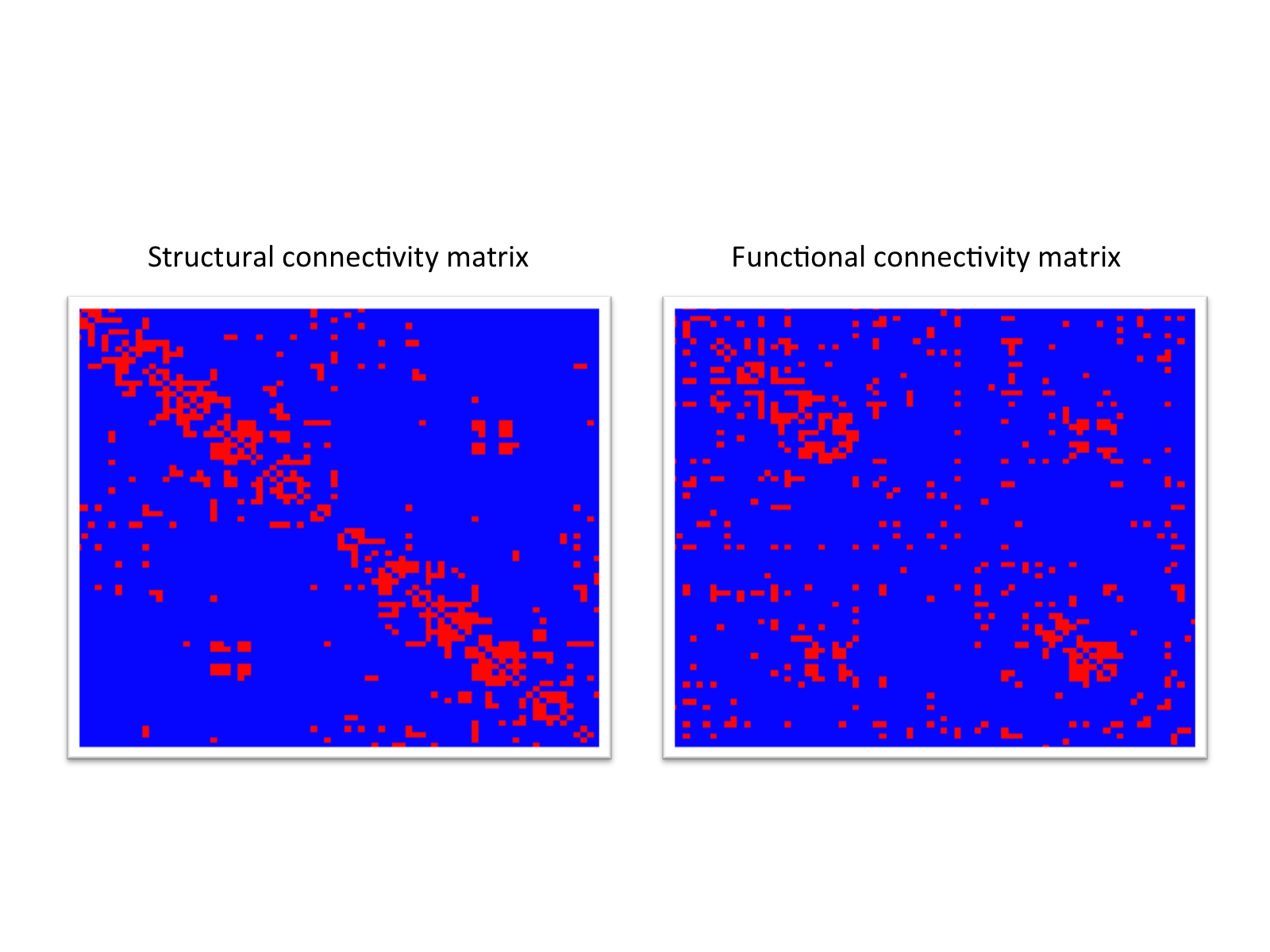

Supplement: Figure S2 — Relation between structural and functional connectivity. Left panel: matrix of the structural connections between all 78 cortical regions, adapted from Gong et al. [24]. Red squares indicate the presence of a connection. Since all connections are bidirectional, the matrix is symmetrical over its diagonal axis. Right panel: matrix of functional connections acquired using the synchronization likelihood (SL) as coupling measure (broadband frequency range: 0.5–45 Hz), and thresholding all pairwise SL values to obtain a graph with the same average degree (K = 8) as the structural connectivity matrix to the left. (TIFF) [file pcbi.1002582.s002.tiff]

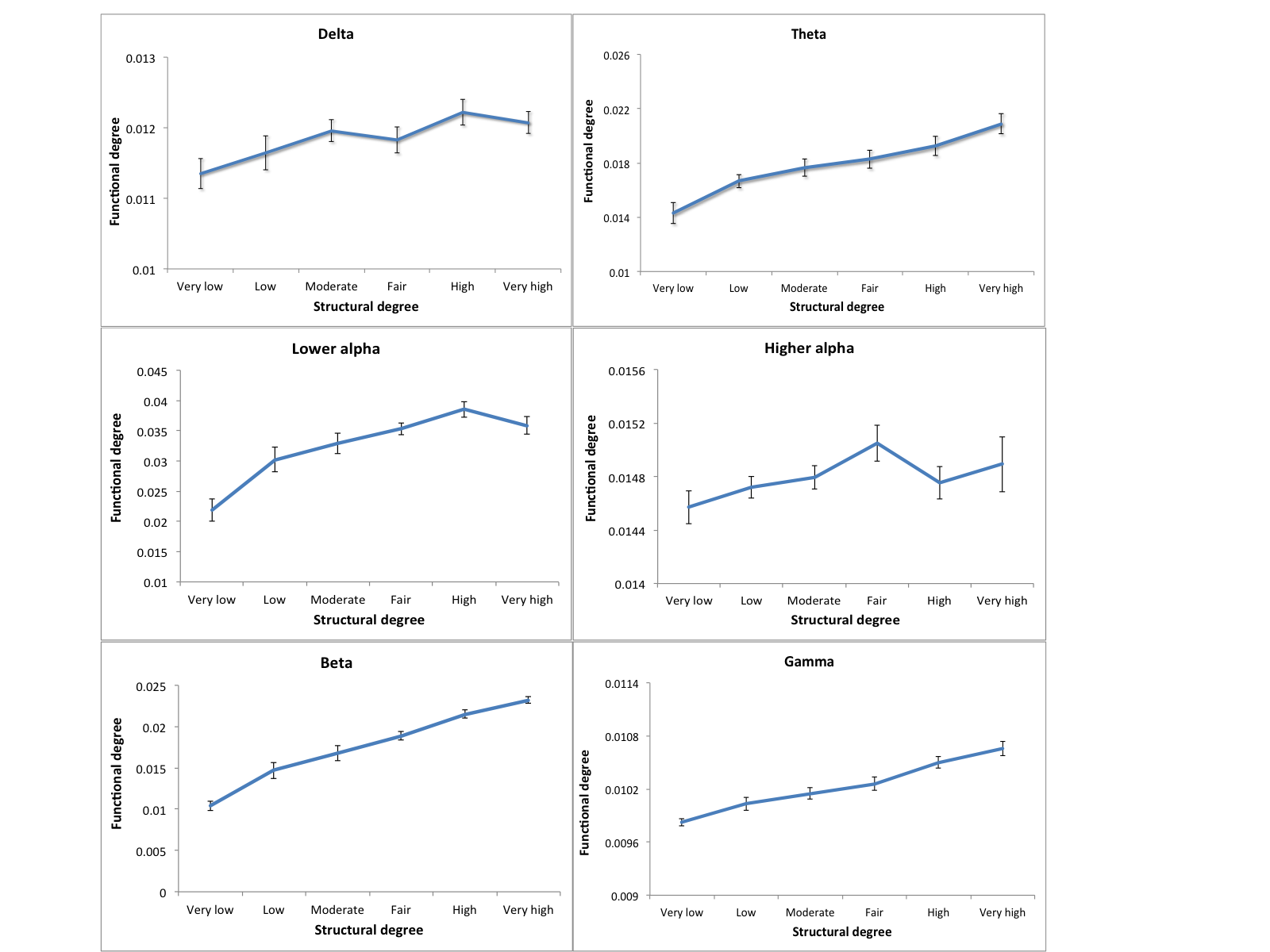

Supplement: Figure S3 — Relation between structural and functional degree in all frequency bands. Error bars depict standard deviations within each bin after 20 simulated runs. (TIF) [file pcbi.1002582.s003.tif]

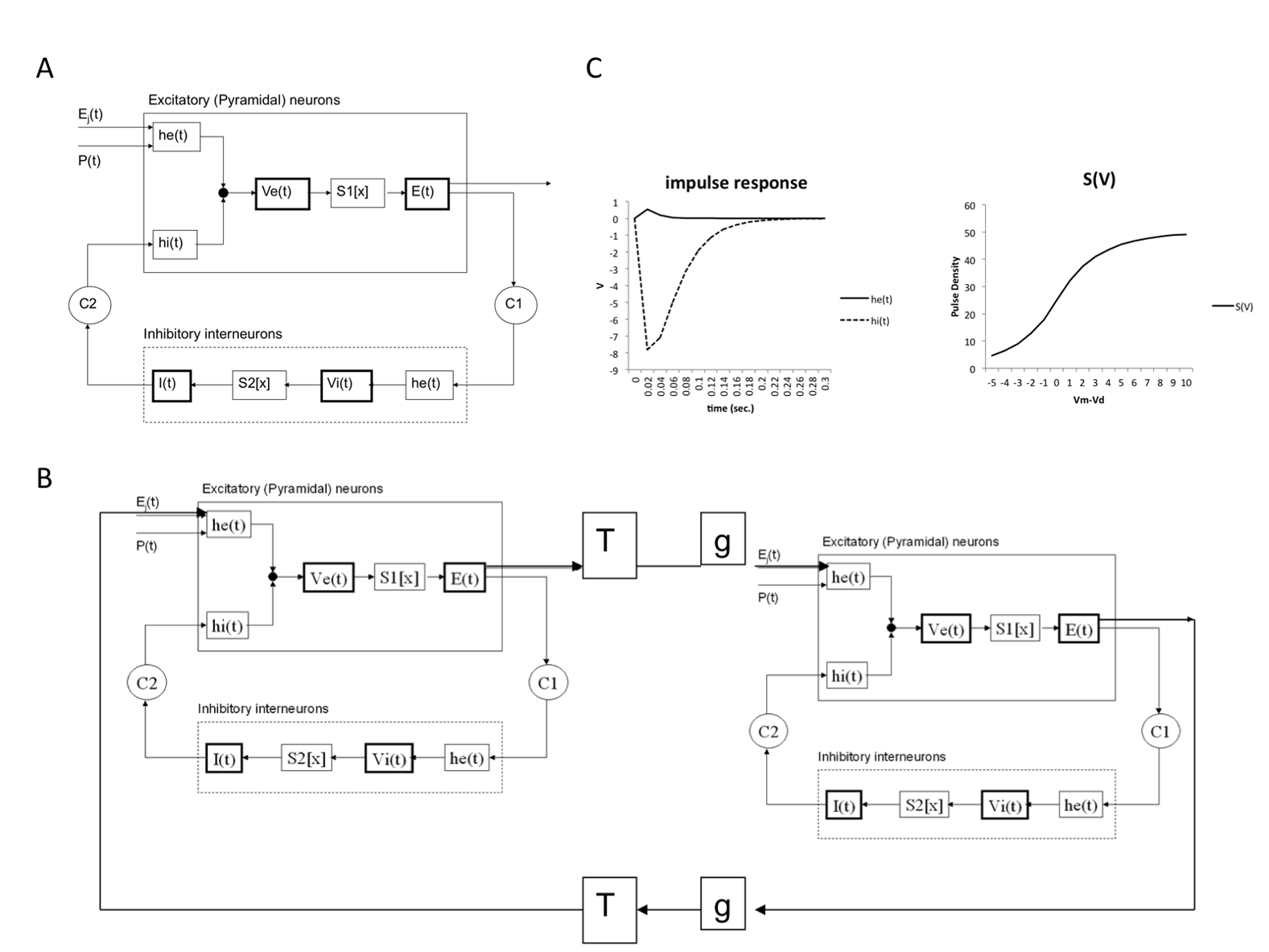

Supplement: Figure S4 — Specifications of the neural mass model. A: Schematic presentation of single neural mass model. The upper rectangle represents a mass of excitatory neurons, the lower rectangle a mass of inhibitory neurons. The state of each mass is modeled by an average membrane potential [Ve(t) and Vi(t)] and a pulse density [E(t) and I(t)]. Membrane potentials are converted to pulse densities by sigmoid functions S1[x] and S2[x]. Pulse densities are converted to membrane potentials by impulse responses he(t) and hi(t). C1 and C2 are coupling strengths between the two populations. P(t) and Ej(t) are pulse densities coming from thalamic sources or other cortical areas respectively. B: Coupling of two neural mass models. Two masses are coupled via excitatory connections. These are characterized by a fixed delay T and a strength g. C: Essential functions of the model. The upper left panel shows the excitatory [he(t)] and inhibitory [hi(t)] impulse responses of Eq. 1. The upper right shows the sigmoid function relating average membrane potential to spike density (Eq. 2). (TIF) [file pcbi.1002582.s004.tif]

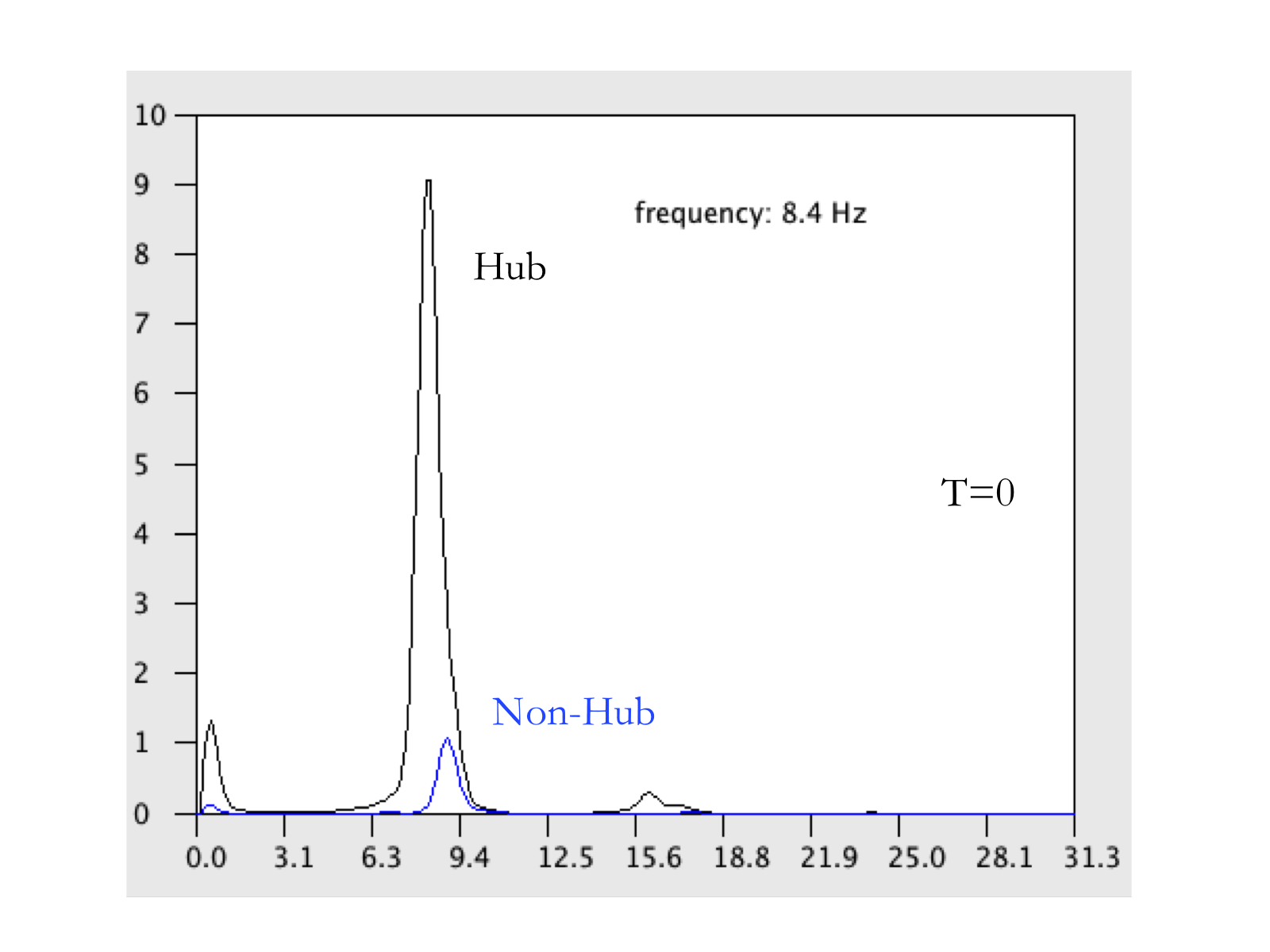

Supplement: Figure S5 — Power spectrum of hubs. Power spectrum of a hub region (precuneus) in black, and a non-hub region in blue. Note the difference in power, but also the lower alpha peak of the hub region. (TIFF) [file pcbi.1002582.s005.tiff]

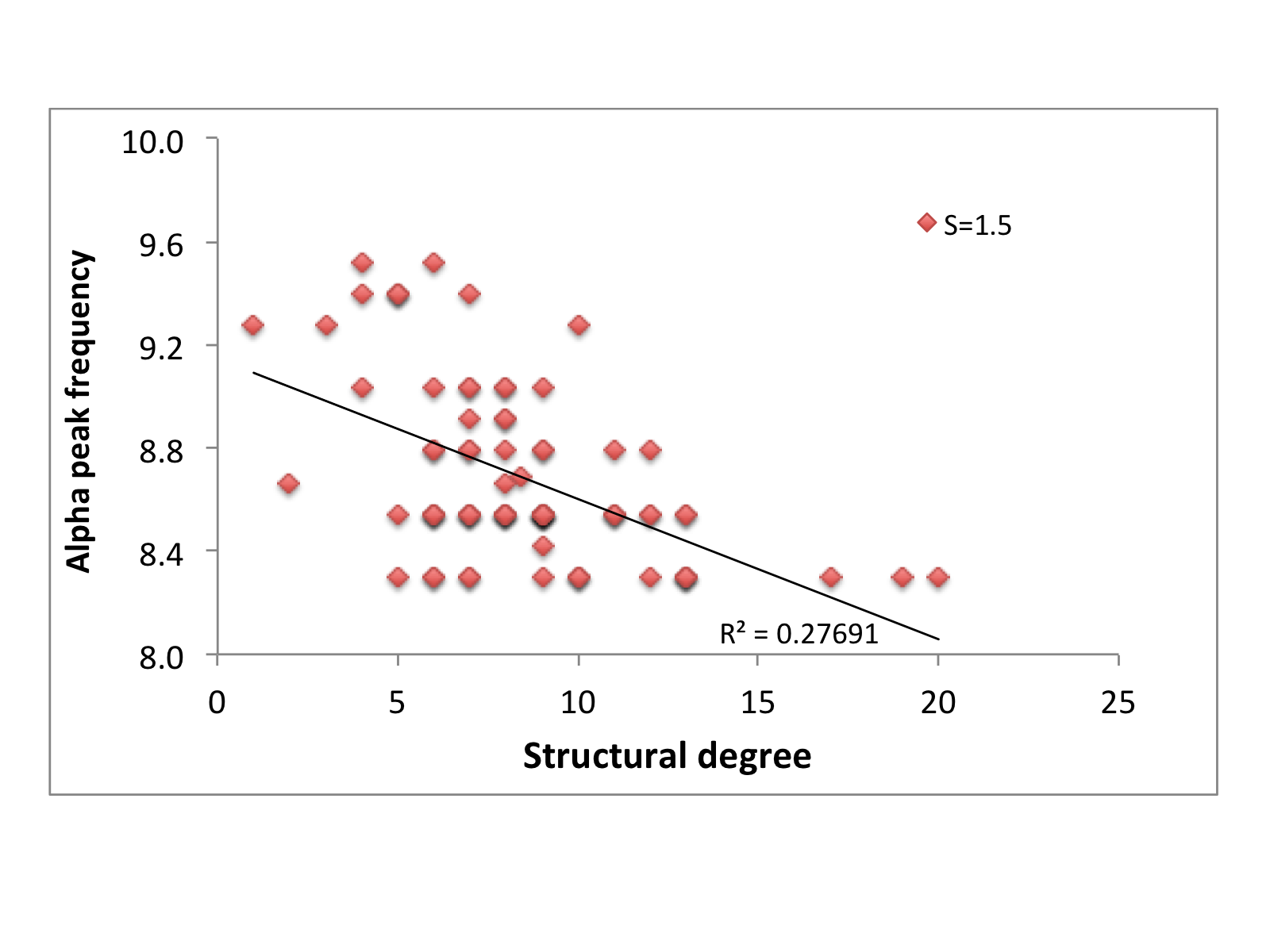

Supplement: Figure S6 — Alpha peak frequency in hubs. The alpha peak frequency of all cortical regions plotted against their structural degree. A negative correlation can be observed (r = −0.53). Hubs (the 13 regions with highest structural degree) have a significantly lower alpha peak (p<0.001) compared to non-hubs. (TIFF) [file pcbi.1002582.s006.tiff]

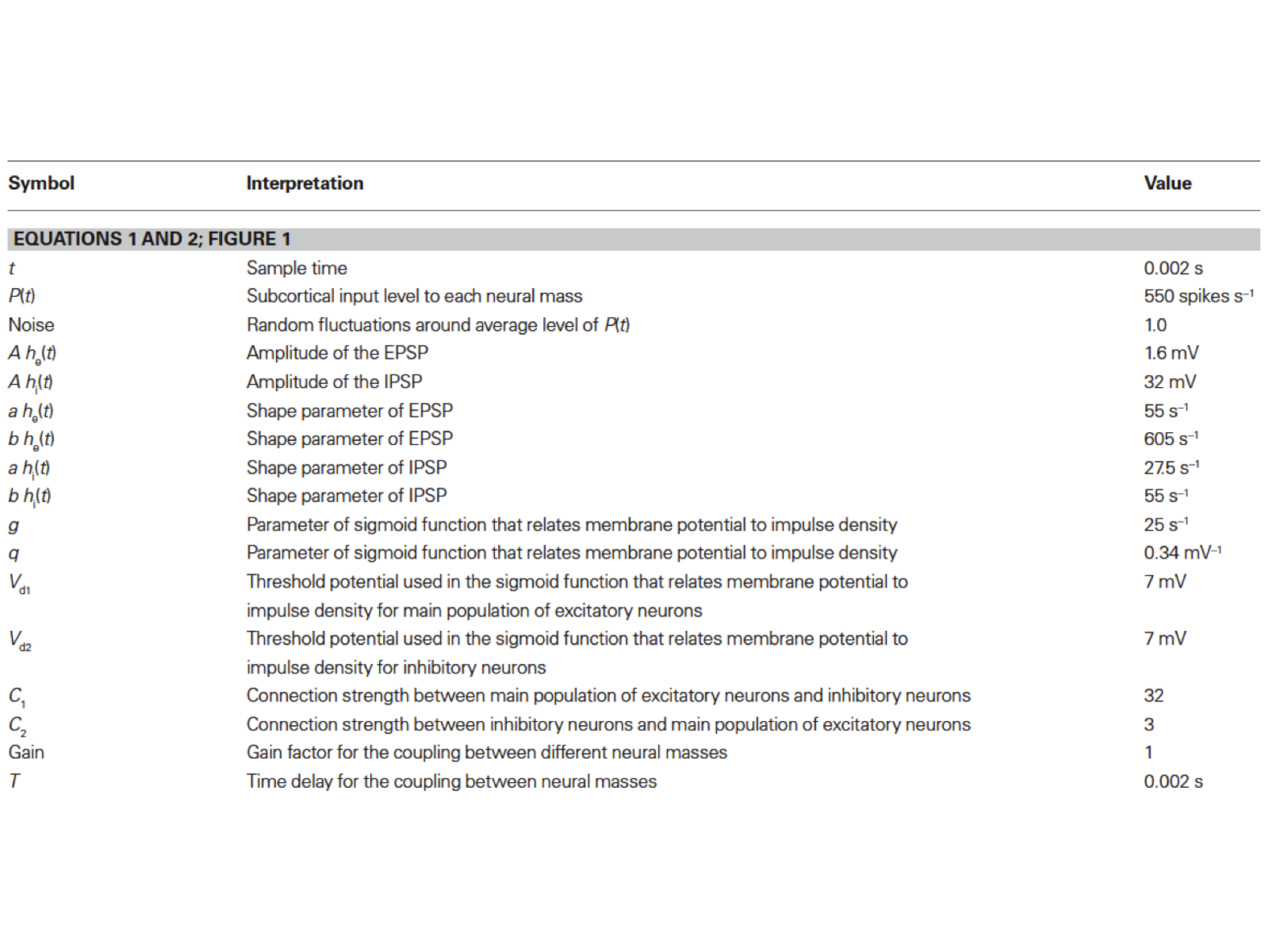

Supplement: Table S1 — Overview of model parameters. The final model consisted of 78 of the NMMs as described above, which were coupled together based on the structural DTI network results from Gong et al. [24]. Coupling between two NMMs, if present, was always reciprocal, and excitatory. The output E(t) of the main excitatory neurons of one NMM was used as the input for the impulse response he(t) of the excitatory neurons of the second NMM; the output E(t) of the second module was coupled to the impulse response he(t) of the excitatory neurons of the first NMM. Following Ursino et al. [87] we used a time delay (T×sample time, with n an integer, 0<T<21) and a gain factor. In the present study, n and gain were set to 1 for all connections. A schematic illustration of the coupling between two NMMs is shown in Figure 1B. For the present study the model was extended in order to be able to deal with activity dependent degeneration of connection strength between multiple coupled NMMs. Coupling strength between neural masses was initially set at the same level for all connections; different levels were tested (S = 1, S = 1.5, S = 2; see figure 3). (TIF) [file pcbi.1002582.s007.tif]
