# Supplementary material for: Activity Dependent Degeneration Explains Hub Vulnerability in Alzheimer's Disease
Source: PLoS Comput Biol. 2012 Aug 16;8(8):e1002582. doi: 10.1371/journal.pcbi.1002582 (PMC3420961; doi:10.1371/journal.pcbi.1002582)
Supplement: Text S1 — Supporting information. 1. Relation between functional degree and total power. 2. Relation between structural and functional degree. 3. Network dynamics: the neural mass model. 4. Relation between structural degree and alpha power peak frequency. (DOC) [file pcbi.1002582.s008.doc]

**Supporting Information**

1. Relation between functional degree and total power
2. Relation between structural and functional degree
3. Network dynamics: the neural mass model
4. Relation between structural degree and alpha power peak frequency

Reference list

1. *Relation between functional degree and total power*

Since activity level might also be influenced by a node’s functional role rather than its structural connectivity status, we performed direct comparisons between functional degree (sum of all weighted *functional* connections of a node) and total power in the common frequency bands (see figure S1). Except for delta and higher alpha, most bands showed strong positive correlations. The beta band showed a remarkably strong correlation (r=0.96). These results suggest that functional hub regions generally have high neuronal activity levels as well, as would be expected intuitively.

*2 Relation between structural and functional degree*

Before examining the relationship between connectivity and activity, we wished to assess if structural and functional connectivity in our brain model was related; i.e. if structural hubs could also be characterized as functional hubs. Therefore, global functional network properties of model simulation data were first investigated. Functional networks all showed small-world organization and weak to moderate modularity in all frequency bands, resembling human MEG data (not shown) [1,12]. Subsequently, we visually compared the structural and broadband functional connectivity matrices (see figure S2). Although not identical, the two matrices evidently share similar connectivity patterns.

To quantify the relation between network structure and function we compared the structural and functional degree for all regions in different frequency bands (see figure S3). In all bands a positive correlation can be observed, indicating that structural hubs are also functional hubs in those frequency ranges. The strongest relation was found in the beta band (r = 0.85, p < 0.001).

*3 Network dynamics: the Neural Mass Model*

We used a model of interconnected neural masses, where each neural mass represents a large population of connected excitatory and inhibitory neurons generating an EEG or MEG like signal. The model was described in Ponten et al. and Stam et al. [3,4]. The basic unit of the model is a neural mass model (NMM) of the alpha rhythm [5,6]. The same model was used in a previous study on bifurcation phenomena of the alpha rhythm [7]. As previously described, this model considers the average activity in relatively large groups of interacting excitatory and inhibitory neurons. Spatial effects are ignored in this model; we will introduce topological effects by coupling several NMMs together. The excitatory and inhibitory populations of each NMM are characterized by their average membrane potentials *V*_e_(*t*) and *V*_i_(*t*), and by their pulse densities, i.e., the proportion of cells firing per unit time *E*(*t*) and *I*(*t*). Static non-linear functions *S*_E_(*x*) and *S*_I_(*x*) relate the potentials *V*_e_(*t*) and *V*_i_(*t*) to the corresponding pulse densities *E*(*t*) and *I*(*t*). The excitatory post-synaptic potential (EPSP) and inhibitory post-synaptic potential (IPSP) are modeled by the impulse responses *h*_e_(*t*) and *h*_i_(*t*). The constants *C*_1_ and *C*_2_ describe the coupling from excitatory to inhibitory and from inhibitory to excitatory populations respectively. *P*(*t*) is the pulse density of an input signal to the excitatory population. Following Zetterberg et al. [6] the following impulse responses were used:


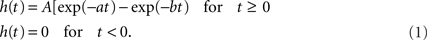


For *h*_e_(*t*) the parameter values were: *A* = 1.6 mV, *a* = 55 s^−1^, *b* = 605 s^−1^. For *h*_i_(*t*) the parameter values were: *A* = 32 mV, *a* = 27.5 s^−1^, *b* = 55 s^−1^. The sigmoid function relating the average membrane potential, *V*_m_, to the impulse density was also taken from Zetterberg et al. [6]:


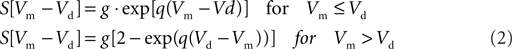


Here the parameter values used were: *q* = 0.34 mV^−1^, *V*_d_ = 7 mV, *g* = 25 s^−1^. For the coupling constants we used *C*_1_ = 32 and *C*_2_ = 3 [5]. A schematic representation is shown in Figure S4A. The activity (spiking rate and power) of the excitatory population in an NMM is largely determined by excitatory input (from the thalamus, but also from other NMMs). All model parameters are summarized in Table S1. The impulse response and sigmoid functions are shown in Figure S4C.

The average membrane potential of the excitatory neurons *V*_e_(*t*) of each of the NMMs separately was the multichannel output. The sample frequency was 500 Hz. In the present study each run consisted of 4096 samples (±8 s). Amount of data selected for analysis was based on previous studies and literature on reproducibility of graph theoretical results [9,10]. The adjacency matrix at the end of each run was subjected to topographical analysis. Table 1 gives an overview of model parameters and initial settings. These parameters go back to a large number of studies with this lumped model, and ultimately to the original model of Lopes da Silva [5].

For the present study the model was extended in order to be able to deal with activity dependent evolution of connection strength between multiple coupled NMMs. Activity dependent degeneration (ADD) was realized by lowering the ‘synaptic’ coupling strength as a function of the spike density of the main excitatory neurons. For each neural mass the spike density of the main excitatory population is stored in a memory buffer that contains the firing rates of the last 20 steps in the model. Step size depends on the sample frequency. At each new iteration, the highest spike density value of the last 20 sample steps is determined and designated as maxAct. From maxAct a loss is determined according to the following relation:

$loss=\exp^{-0.01\max Act}$ (3)

Since maxAct is non-negative, loss will be a number between 0 and 1. Next, the coupling values C1 (connections between main excitatory population and inhibitory population), C2 (connections between inhibitory population and main excitatory population), Pt (thalamic input to main excitatory population) and S (*structural* coupling strength between neural masses) are all multiplied by loss to obtain their new lower values.

The model was programmed in Java and implemented in the program BrainWave (version 0.9.04, written by C.J. Stam, available on home.kpn.nl/stam7883). The Java code was based on the Pascal source code described by Schuuring [11].

4 *Relation between structural degree and alpha peak frequency*

An additional finding in our experiments is that hub regions have a slightly *lower* alpha peak in their power spectrum, as is shown in figures S5 and S6.

It might be possible that a high structural connectivity slows regions down, however this finding should be verified in neurophysiological experiments. NB: the mu rhythm, which is a variation of the alpha rhythm that is present in cortical regions that are not seen as hub regions, is indeed faster than the dominant posterior alpha rhythm.

**Reference list**

1. Stam CJ, de Haan W, Daffertshofer A, Jones BF, Manshanden I, et al (2009) Graph theoretical analysis of magnetoencephalographic functional connectivity in Alzheimer's disease. Brain 132: 213-224.

2. Gong G, He Y, Concha L, Lebel C, Gross DW, et al (2009) Mapping anatomical connectivity patterns of human cerebral cortex using in vivo diffusion tensor imaging tractography. Cereb Cortex 19: 524-536.

3. Ponten SC, Daffertshofer A, Hillebrand A, Stam CJ (2010) The relationship between structural and functional connectivity: graph theoretical analysis of an EEG neural mass model. Neuroimage 52: 985-994.

4. Stam CJ (2010) Characterization of anatomical and functional connectivity in the brain: a complex networks perspective. Int J Psychophysiol 77: 186-194.

5. Lopes da Silva FH, Hoeks A, Smits H, Zetterberg LH (1974) Model of brain rhythmic activity. Biological Cybernetics 15: 27-37.

6. Zetterberg LH, Kristiansson L, Mossberg K (1978) Performance of a model for a local neuron population. Biological cybernetics 31: 15-26.

7. Stam CJ, Pijn JP, Suffczynski P, Lopes da Silva FH (1999) Dynamics of the human alpha rhythm: evidence for non-linearity? Clin Neurophysiol 110: 1801-1813.

8. Ursino M, Zavaglia M, Astolfi L, Babiloni F (2007) Use of a neural mass model for the analysis of effective connectivity among cortical regions based on high resolution EEG recordings. Biological cybernetics 96: 351-365.

9. Deuker L, Bullmore ET, Smith M, Christensen S, Nathan PJ, et al (2009) Reproducibility of graph metrics of human brain functional networks. Neuroimage 47: 1460-1468.

10. Whitlow CT, Casanova R, Maldjian JA (2011) Effect of resting-state functional MR imaging duration on stability of graph theory metrics of brain network connectivity. Radiology 259: 516.

11. Schuuring D (1988) Modelleren en simuleren van epileptische activiteit. Master thesis, University of Twente, the Netherlands.

12. de Haan W, van der Flier WM, Koene T, Smits LL, Scheltens P, Stam CJ (2011)

Disrupted modular brain dynamics reflect cognitive dysfunction in Alzheimer's disease. Neuroimage Nov 30. [Epub ahead of print]
